# Altered functional connectivity in inter-effector and effector-specific subregions of the primary motor cortex in Parkinson's disease

**DOI:** 10.1016/j.nicl.2026.104051

**Published:** 2026-08-26

**Authors:** Huazhi Li, Wei Chen, Yapeng Qi, Wenshuang Tang, Chao Zhang, Hao Fan, Liang Shu, Lipeng Lin, Jian-Ren Liu, Xiaoxia Du

**Affiliations:** a School of Psychology, Shanghai University of Sport, Shanghai 200438, China; b Department of Neurology, Shanghai Ninth People's Hospital, Shanghai Jiao Tong University School of Medicine, Shanghai, China; c Clinical Research Center, Shanghai Jiao Tong University School of Medicine, Shanghai, China; d Center for Exercise and Brain Science, Shanghai University of Sport, Shanghai, China, 200438

**Keywords:** Parkinson's disease, Primary motor cortex, Functional connectivity, Resting-state fMRI, Motor cortex subregions

## Abstract

**Objectives:**

Parkinson's disease (PD) involves large-scale network disruptions, yet functional connectivity (FC) alterations specific to distinct primary motor cortex (M1) subregions and their subcortical targets remain poorly characterized. We aimed to investigate subregion-specific resting-state FC alterations in PD and their clinical associations.

**Methods:**

Resting-state fMRI data from 37 PD patients and 37 matched healthy controls were analyzed. Seed-to-voxel and expanded ROI-to-ROI analyses (incorporating subcortical nuclei) mapped FC patterns of two M1 subregions: inter-effector and effector-specific. A System Segregation index quantitatively assessed large-scale network boundaries.

**Results:**

PD patients exhibited local intra-M1 hyperconnectivity and subsystem-specific cortico-subcortical hyperconnectivity, particularly involving the putamen and thalamus for the effector-specific region. Seed-to-voxel analyses revealed divergent long-range dysconnectivity: the inter-effector region showed hypoconnectivity with sensorimotor and limbic areas, whereas the effector-specific region exhibited sensorimotor hypoconnectivity and a pathological loss of anti-correlation with cerebellar and frontoparietal networks. Quantitative evaluation confirmed a significant collapse in the System Segregation index, validating network boundary breakdown. Clinically, inter-effector hypoconnectivity to the right Rolandic operculum correlated with freezing of gait severity, while aberrant effector-specific-cerebellar positive FC correlated with tremor severity.

**Conclusions:**

This study delineates a multilayered network pathology in PD, involving local hyperconnectivity, cortico-subcortical desegregation, and quantitatively verified impairment of large-scale network segregation. These subregion-specific alterations, particularly maladaptive cortico-cerebellar and cortico-striatal coupling, reframe PD as a disorder of network dedifferentiation, highlighting M1 subcircuits as targets for symptom-specific neuromodulation.

## Highlights

• M1 subregions show distinct connectivity profiles in Parkinson's disease.
• Local hyperconnectivity emerges between motor cortex subregions in patients.
• Maladaptive motor-cerebellar coupling correlates with tremor severity.
• Inter-effector network isolation is linked to freezing of gait.

## Graphical abstract

**Figure:**
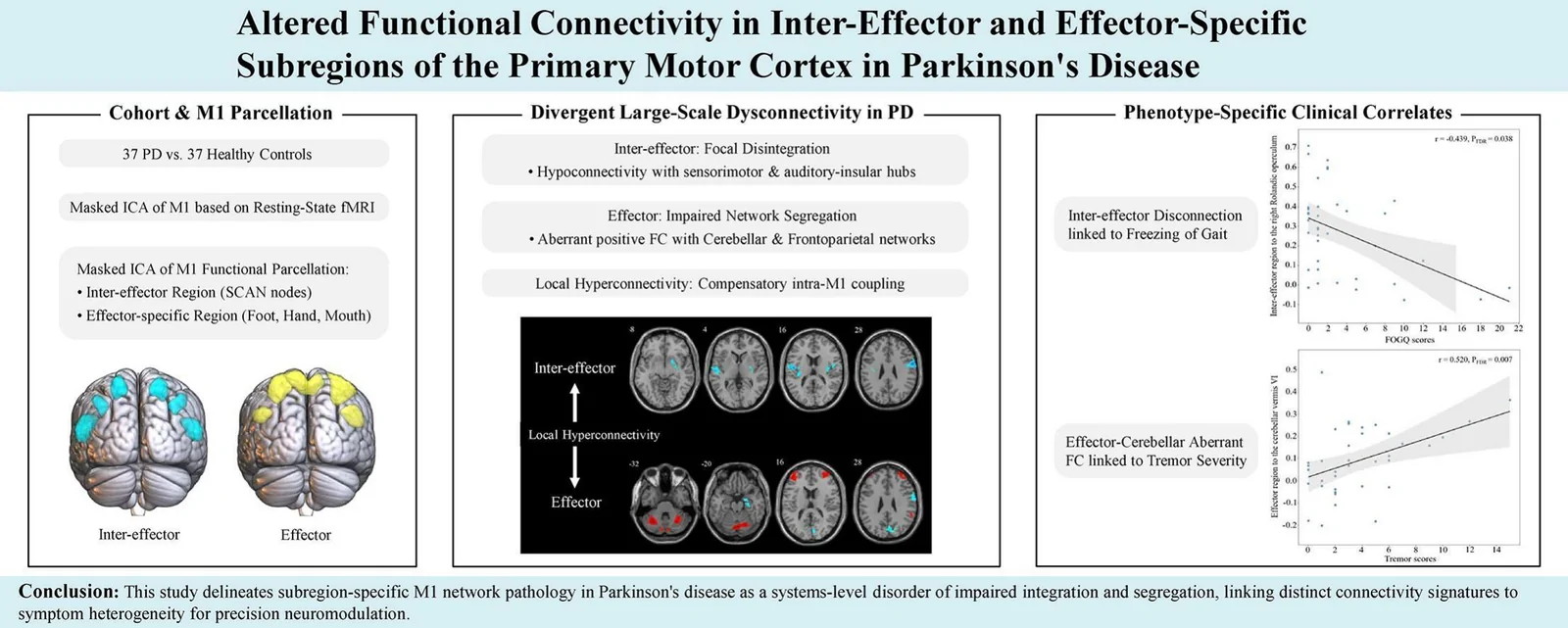

## 1 Introduction

Parkinson's disease (PD) is the second most common neurodegenerative disorder, characterized by heterogeneous motor and non-motor symptoms that pose substantial diagnostic challenges (Armstrong and Okun, 2020; Tolosa et al., 2021). Although classically defined by striatal dopaminergic depletion, this model does not fully account for the broad clinical spectrum of PD, which increasingly implicates dysfunction across multiple large-scale brain networks (Armstrong and Okun, 2020; Ji et al., 2018).

The primary motor cortex (M1), traditionally regarded as the final cortical region responsible for motor execution, has a complex functional architecture that is increasingly linked to the pathophysiology of PD. Recent pioneering research has reinterpreted M1 as comprising two interconnected systems: (1) inter-effector regions that constitute a somato-cognitive action network (SCAN) (Gordon et al., 2023), and (2) effector-specific regions that govern precise, isolated movements of individual body parts (e.g., foot, hand, mouth). The SCAN plays a crucial role in planning integrated whole-body actions, axial motor control, and the integration of physiological states—functions that are markedly impaired in PD. Crucially, recent multimodal evidence has fundamentally reframed PD as a “SCAN disorder”, revealing that pathological hyperconnectivity from basal ganglia loops is selectively directed toward these cortical SCAN nodes rather than effector-specific regions (Ren et al., 2026). This novel parcellation provides a new anatomical framework for understanding PD symptoms: alterations in effector-specific circuits may result in fine motor dysfunctions such as bradykinesia and tremor, whereas dysfunction within the SCAN and its interactions with higher-order networks (e.g., the cingulo-opercular network (CON)) may account for axial symptoms, postural instability, autonomic disturbances, and challenges in action initiation (Bohnen et al., 2023; Gordon et al., 2023).

Resting-state functional magnetic resonance imaging (rs-fMRI) has been instrumental in delineating extensive alterations in functional connectivity (FC) across PD networks, including the sensorimotor, cerebellar, and default mode networks (Tessitore et al., 2019). Convergent evidence from electrophysiology, neurostimulation, and neuroimaging affirms that the functional activity and physiology of M1 are markedly altered in PD, thereby significantly contributing to motor signs (Burciu and Vaillancourt, 2018; Underwood and Parr-Brownlie, 2021). These alterations are manifested as changes in movement-related neuronal dynamics, reorganization of local circuits, pathological beta oscillations, and persistent functional disturbances observable even at rest (Burciu and Vaillancourt, 2018; Underwood and Parr-Brownlie, 2021). Nonetheless, neuroimaging research has predominantly regarded M1 as a homogeneous entity. Consequently, despite acknowledging M1 as a critical node in the pathophysiology of PD (Underwood and Parr-Brownlie, 2021), previous studies have not systematically applied this modern, functionally graded parcellation to investigate disease-related modifications in brain organization. This lacuna in the existing literature raises vital questions: Are the FC within and between these M1 subregions, along with their dedicated cortico-subcortical pathways and connections with other large-scale networks, selectively impaired? Most importantly, how do these unique patterns of subregional dysfunction correlate with the diverse and progressive clinical features of PD?

This study aims to address these questions by using rs-fMRI to delineate the M1 into inter-effector and effector subregions and to examine associated neural abnormalities in PD across multiple levels. Specifically, the investigation will assess: (1) FC within and between the identified M1 subregions, as well as their targeted functional coupling with core basal ganglia-thalamic nuclei; and (2) whole-brain FC patterns initiated from each subregion. These FC metrics will be correlated with comprehensive motor and non-motor clinical assessments in patients diagnosed with PD.

By systematically applying this refined functional parcellation model to the intrinsic organization of M1, this study seeks to extend the emerging framework of PD as a “SCAN disorder” beyond subcortical circuitry. We expect that our results will elucidate the complex spatial organization of motor cortex dysfunction in PD and offer innovative, network-based insights into the neural substrates of its diverse clinical manifestations. Ultimately, this research may contribute to the development of targeted therapeutic strategies to modulate the brain circuits underlying specific symptoms.

## 2 Methods

### 2.1 Subjects

Patients with idiopathic PD were recruited from the Neurology Department of Shanghai Ninth People's Hospital between November 2017 and January 2021. Inclusion criteria were: (a) diagnosis of PD meeting the 2015 Movement Disorder Society (MDS) clinical diagnostic criteria (Postuma et al., 2015); (b) age between 40 and 80 years; (c) right-handedness and native Mandarin Chinese speakers; (d) Hoehn and Yahr (H—Y) stage I–III; (e) Mini-Mental State Examination (MMSE) score > 24; and (f) ability to complete all assessments. Exclusion criteria were: (a) MRI contraindications or inability to tolerate scanning; and (b) presence of other neurological disorders (e.g., dementia, stroke) or severe psychiatric illnesses.

Experienced neurologists collected demographic and clinical data. Disease severity was rated using the H—Y stage and the MDS-Unified Parkinson's Disease Rating Scale Part III (MDS-UPDRS-III). A Tremor Score was derived from specific MDS-UPDRS-III items (items 3.15–3.18). Freezing of gait was assessed with the Freezing of Gait Questionnaire (FOGQ). Non-motor symptoms were evaluated using the MMSE, the Montreal Cognitive Assessment-Basic (MoCA-B), and the 17-item Hamilton Depression Rating Scale (HAMD-17). All patients were assessed in the clinically defined “ON” state for medication. The Levodopa Equivalent Daily Dose (LEDD) was calculated for each patient (Jost et al., 2023).

A group of age- and sex-matched healthy controls (HCs) was also recruited. HC inclusion criteria were: (a) age between 40 and 80 years; (b) right-handed, native Mandarin Chinese speakers; and (c) no cognitive impairment or history of psychiatric disorders. Exclusion criteria were the same as those for PD patients, with the addition of any significant chronic systemic illness.

Participants were sequentially excluded from the initial recruited cohort based on the following criteria: (1) incomplete clinical assessments or missing imaging data (13 patients with PD, 7 HCs); (2) failure to achieve strict age and sex matching between the PD and HC groups (8 patients with PD, 11 HCs); and (3) excessive head motion during the rs-fMRI scan, defined as a mean framewise displacement (mFD) ≥ 0.2 mm (22 patients with PD, 24 HCs). Following this rigorous quality control and matching process, the final cohort comprised 37 patients with PD and 37 HCs. Notably, the age range of the final included sample (49–73 years) was not a predefined restriction but rather the empirical result of this stringent exclusion cascade. All statistical analyses, including demographic comparisons (independent-samples *t*-tests and chi-square tests) and subsequent clinical correlation analyses, were performed using R software (version 4.4.2).

### 2.2 MRI acquisition

Imaging was performed on a Siemens Prisma 3.0 T MRI scanner with a 64-channel head coil at the Shanghai Key Laboratory of Magnetic Resonance. Head motion was minimized using foam padding. The protocol included:

T1-weighted structural images: Acquired using a magnetization-prepared rapid gradient-echo (MPRAGE) sequence (TR = 2530 ms, TE = 2.98 ms, TI = 1100 ms, FA = 7°, 192 sagittal slices, voxel size = 1 × 1 × 1 mm^3^).

Rs-fMRI: Acquired using a T2-weighted echo-planar imaging (EPI) sequence (TR = 2000 ms, TE = 30 ms, FA = 90°, 33 axial slices, voxel size = 3.5 × 3.5 × 3.5 mm^3^, 240 volumes). Participants were instructed to keep their eyes closed, remain still, and stay awake.

### 2.3 Data preprocessing

The rs-fMRI data were preprocessed using the CONN toolbox (v.22.0). The first 10 volumes were discarded for signal equilibrium. Standard steps included slice-timing correction, realignment, outlier detection (ART toolbox), co-registration to individual T1-weighted images, spatial normalization to the Montreal Neurological Institute (MNI) space, and smoothing with a 6-mm full-width-at-half-maximum Gaussian kernel.

Denoising was performed by regressing out: (1) five principal components each from white matter and cerebrospinal fluid signals (aCompCor); (2) 12 motion parameters (rigid-body estimates and their first-order derivatives); and (3) linear trends. Volumes with FD > 0.5 mm or global signal changes >3 standard deviations were scrubbed. Subjects with a mean FD ≥ 0.2 mm were excluded. A band-pass filter (0.008–0.09 Hz) was applied to retain low-frequency fluctuations (Hallquist et al., 2013).

### 2.4 Independent component analysis and region of interest (ROI) definition

Group-level independent component analysis (ICA) was conducted in CONN (Calhoun et al., 2001) using the pooled rs-fMRI data from all participants. To ensure mathematical symmetry and avoid “template bias” that could arise from applying a healthy control (HC)-only spatial template to brains with PD-related atrophy and functional reorganization, the Group ICA was deliberately performed on the pooled cohort (PD and HC). This approach establishes an unbiased consensus space, while the GICA3 back-reconstruction algorithm preserves highly individualized spatial maps for each subject (Erhardt et al., 2011). Building on prior research that used approximately 10 components to delineate brain networks, and further supported by our evaluation of IC numbers ranging from 10 to 30 to ensure robust M1 identification, we set the number of independent components (ICs) to 10. The data were initially subjected to principal component analysis (PCA) for dimensionality reduction, followed by the fast-ICA algorithm and GICA3 back-projection to obtain subject-specific components. The M1 network was identified by visual inspection and a spatial correlation (*r* = 0.385) with the precentral gyrus mask from the Automated Anatomical Labeling atlas 3 (AAL3) (Rolls et al., 2020). This correlation value reflects the intrinsic nature of the sensorimotor network, which functionally extends beyond the strict macroscopic boundaries of the precentral gyrus to encompass adjacent co-activated regions (e.g., the postcentral gyrus and supplementary motor area), thereby preserving the network's authentic physiological topology rather than artificially truncating it.

**Supplementary Fig. S1:**
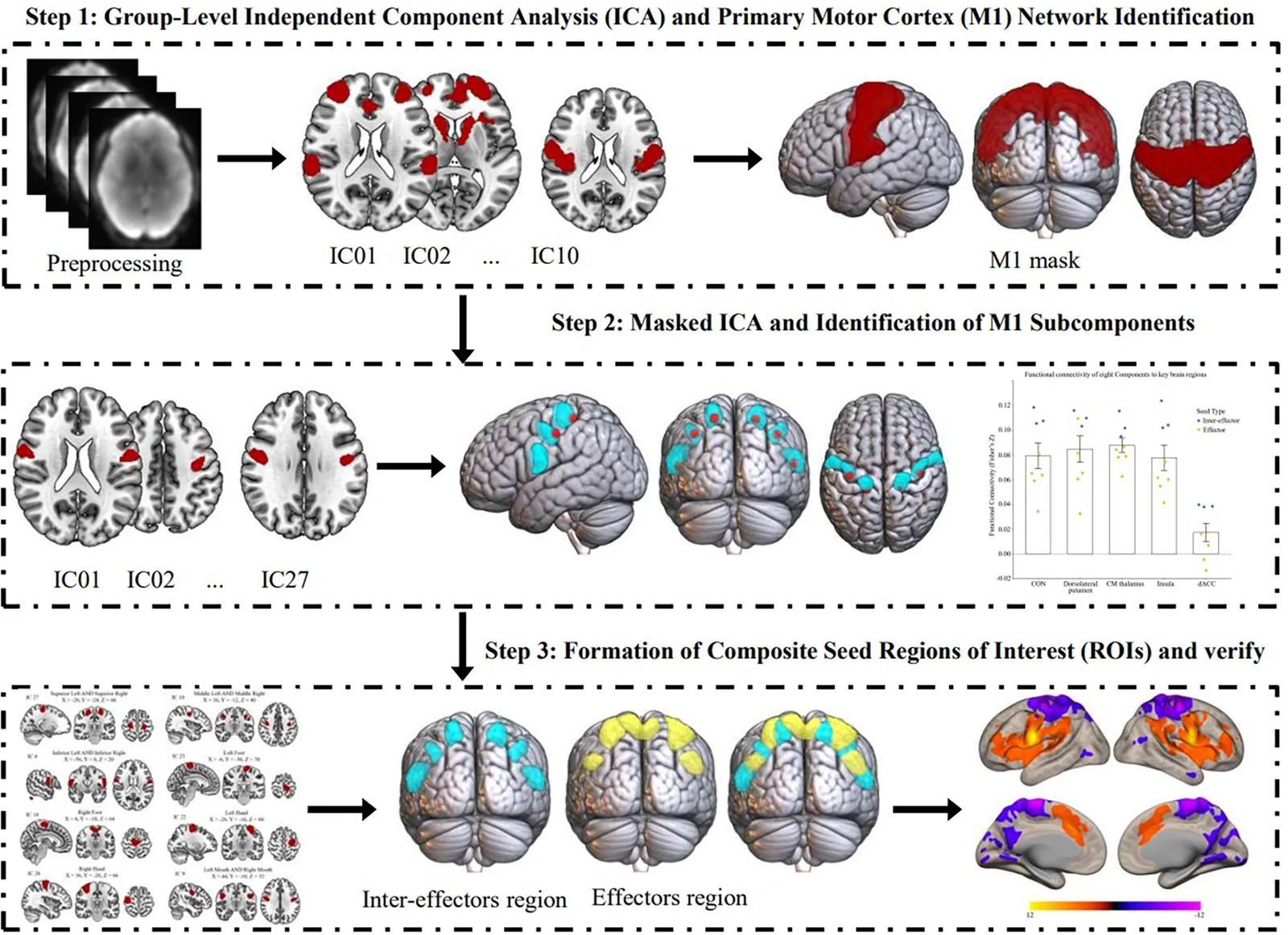
Flowchart illustrating the three-stage analytical pipeline for defining global inter-effector and effector seed regions within the primary motor cortex (M1). Critically, this parcellation was conducted at the cohort level—simultaneously across all participants, with patients and controls pooled—thereby ensuring that a single, consistent functional boundary set was derived from the whole sample. Stage 1 (Group-Level ICA & M1 Network Identification): Preprocessed group fMRI data were analyzed with the number of independent components (ICs) set to 10, a parameter confirmed to produce a reliable M1 component after testing ICs ranging from 10 to 30. The data were processed using principal component analysis (PCA) for dimensionality reduction, followed by the fast-ICA algorithm and GICA3 back-projection to generate subject-specific components. The M1 network was identified visually and through a high spatial correlation (r = 0.385) with an anatomical mask of the precentral gyrus (AAL3 atlas). The resulting component map was binarized to create an M1 mask. Stage 2 (Masked ICA & M1 Subcomponent Identification): A masked ICA (mICA) was performed only within the M1 mask, following the same steps (PCA, fast-ICA, GICA3). After testing model stability across 20–40 ICs, a 27-component solution was chosen. Eight components from this set were retained and classified into two categories based on two criteria: 1) the proximity of peak coordinates to previously reported inter-effector locations (represented by red spherical ROIs), and 2) the difference in functional connectivity with the cingulo-opercular network (CON) and key hubs (dorsal anterior cingulate cortex, insula), as well as with the dorsolateral putamen and thalamic centromedian nucleus. This resulted in three inter-effector and five effector-specific subcomponents. Stage 3 (Formation of Composite Seed ROIs and Verification): The subcomponents were merged based on spatial proximity and functional similarity. The three inter-effector subcomponents were combined to create a global inter-effector seed ROI, and the five effector-specific subcomponents were merged into a global effector seed ROI. Whole-brain FC comparison between inter-effector and effector regions in the control group revealed significantly stronger connectivity between the inter-effector region and CON, confirming the segmentation of M1 subregions.

To parcellate M1 into inter-effector and effector subregions, a masked ICA (mICA) was performed within the extracted M1 mask across the entire cohort. Critically, this parcellation was conducted at the cohort level—simultaneously across all participants, with patients and controls pooled—thereby ensuring that a single, consistent functional boundary set was derived from the whole sample. The same analytic steps (PCA, fast-ICA, and GICA3 back-projection) were applied. After testing model order stability across a range of ICs (20–40), a 27-component solution was selected. From this solution, eight components were retained and classified using two criteria: (1) spatial proximity of their peak coordinates to previously reported MNI coordinates of the inter-effector region (Gordon et al., 2023)—quantitatively validated by a short mean Euclidean distance of 4.97 mm, which confirms high spatial homology (node-specific coordinates and distances for the bilateral superior, middle, and inferior ROIs are detailed in Supplementary Table 1); and (2) their differential mean FC with the CON and its key hubs (the dorsal anterior cingulate cortex (dACC) and insula), as well as with the dorsolateral putamen and thalamic centromedian nucleus (CM) (all of which exhibit significantly stronger FC with the SCAN) (Gordon et al., 2023). This procedure yielded three inter-effector components (spanning bilateral superior, middle, and inferior zones) and five effector components (representing left foot, right foot, left hand, right hand, and bilateral mouth). Based on spatial contiguity and functional homology, these components were merged to form two composite seeds: a global inter-effector region and a global effector region. The processing flow is shown in Supplementary Fig. 1.

### 2.5 Functional connectivity and network segregation analyses

Functional connectivity analyses, encompassing both ROI-to-ROI and seed-to-voxel approaches, were performed using the CONN toolbox. To comprehensively evaluate cortico-subcortical network dynamics, the ROI-to-ROI analysis was expanded to include the composite inter-effector and effector seeds, along with six a priori subcortical regions that are structurally and functionally implicated in SCAN and PD pathophysiology (Ren et al., 2026). These targeted subcortical ROIs comprised the substantia nigra (SN), subthalamic nucleus (STN), globus pallidus internus (GPi), globus pallidus externus (GPe), putamen, and the ventral intermediate/centromedian nucleus of the thalamus (VIM/CM), with spatial masks derived from the supplementary materials of Ren et al. (2026). FC strength was quantified using Fisher's *Z*-transformed bivariate correlation coefficients within a weighted general linear model (GLM). Group-level GLMs compared the PD and HC groups, controlling for age and sex.

Direct Statistical Contrast (ANCOVA): To rigorously disentangle shared network alterations from subregion-specific pathologies and provide voxel-wise statistical evidence for subregion dissociation, a whole-brain 2 (Group: PD vs. HC) × 2 (Seed: Inter-effector vs. Effector) mixed analysis of covariance (ANCOVA) was conducted using a flexible factorial design, with age and sex included as covariates of no interest. Significant interaction effects (Group × Seed) were thresholded at a voxel-level *P* < 0.001 and a cluster-level P_FDR_ < 0.05.

ROI-to-ROI Analysis: Significance was determined using the Functional Network Connectivity (FNC) method (Jafri et al., 2008), with thresholds set at a connection-level *P* < 0.05 and a cluster-level *P* < 0.05 with false discovery rate (FDR) correction.

Seed-to-Voxel Analysis: Whole-brain voxel-wise comparisons were thresholded at a voxel-level *P* < 0.001, with cluster-level significance determined by Gaussian Random Field **(GRF)** theory (cluster-*P*_FDR_ < 0.05) (Chumbley et al., 2010). This stringent primary voxel-level threshold was deliberately chosen to effectively constrain the family-wise error rate and mitigate the risk of inflated false positives often associated with modest sample sizes (Eklund et al., **2016****;** Woo et al., **2014****).**

Quantitative Network Segregation: To numerically quantify the large-scale network segregation of M1 subregions, we calculated the subregion-specific System Segregation index, adapting the theoretical framework established by Chan et al. (2014). For each participant, within-system connectivity (Z̄_w_) was defined as the mean Fisher's *Z*-transformed correlation among the internal components of each functionally defined M1 subregion (i.e., the inter-effector or effector composite seeds). Between-system connectivity (Z̄_b_) was calculated as the mean connectivity between the specific M1 subregion and the spatially distinct target clusters identified in the seed-to-voxel analyses. Critically, following the methodology of Chan et al. (2014), all negative correlation values were set to zero prior to averaging. This specific mathematical treatment ensures that the metric strictly isolates and evaluates the pathological intrusion of anomalous positive functional couplings (i.e., the loss of normal anti-correlations). The System Segregation index was then computed as (Z̄w − Z̄b) / Z̄w. Group differences in these indices were assessed using Welch's two-sample *t*-tests.

Clinical Correlation Analysis: Significant FC alterations (extracted as mean eigenvariates from the significant clusters) were further correlated with clinical scores (UPDRS-III, Tremor Score, FOGQ, MMSE, MoCA-B, HAMD-17, and disease duration) using partial correlations, controlling for age, sex, and LEDD (thresholded at *P*_FDR_ < 0.05). To address concerns regarding effect size inflation in modest samples, Hedges' g was calculated and reported for all significant group differences to provide unbiased effect size estimates.

## 3 Results

### 3.1 Demographic and clinical characteristics

Following rigorous data quality control (which excluded 46 participants due to excessive head motion, defined as a mean FD ≥ 0.2 mm) and strict demographic matching, the final study cohort comprised 37 patients with PD and 37 HCs. The resulting age range of the included participants was 49 to 73 years, reflecting the empirical distribution of the retained sample rather than a predefined age cutoff. The two groups did not differ significantly in age (*P* = 0.691) or sex distribution (*P* = 0.816). The PD cohort consisted of 17 patients with left-sided onset, 14 with right-sided onset, and 6 with bilateral involvement. Detailed demographic and clinical information is presented in Table 1.

**Table 1:** Demographic and clinical characteristics of the two groups.

Characteristics (mean ± SD)	PD (*n* = 37)	HC (n = 37)	*P*-value
Age (years)	62.43 ± 6.37	61.84 ± 6.45	0.691
Sex (male/female)	20/17	18/19	0.816
mFD (mm)	0.10 ± 0.03	0.11 ± 0.04	0.504
H-Y	1.71 ± 0.73		
Duration (years)	3.02 ± 3.18		
LEDD (mg)	216.55 ± 260.06		
UPDRS-III	21.38 ± 15.85		
Tremor Score	3.73 ± 3.41		
FOGQ	3.57 ± 4.99		
MMSE	27.81 ± 1.84		
MoCA-B	23.17 ± 3.74		
HAMD-17	6.46 ± 5.94		

Note: SD, standard deviation; PD, Parkinson's disease; HC, healthy control; mFD, mean framewise displacement; H—Y, Hoehn and Yahr stage; LEDD, levodopa equivalent daily dose; UPDRS-III, Movement Disorder Society-Unified Parkinson's Disease Rating Scale-Part III; FOGQ, Freezing of Gait Questionnaire; MMSE, Mini-Mental State Examination; MoCA-B, Montreal Cognitive Assessment-Basic; HAMD-17, 17-item Hamilton Depression Rating Scale.

### 3.2 ROI-to-ROI analysis

Expanded ROI-to-ROI analysis revealed a broad pattern of pathological hyperconnectivity within the cortico-basal ganglia-thalamic network in patients with PD compared to HCs (detailed in Table 2).

**Table 2:** Significant ROI-to-ROI functional connectivity increases (hyperconnectivity) between primary motor cortex (M1) subregions and targeted basal ganglia-thalamic nuclei in patients with Parkinson's disease compared to healthy control.

Subregions	Subcortical nuclei	T-value	Hedges' g	P_FDR_
Inter-Effector	GPi	4.09	0.94	< 0.001
	STN	3.77	0.87	0.001
	GPe	2.52	0.58	0.032
Effector	GPi	4.73	1.09	< 0.001
	STN	4.67	1.07	< 0.001
	GPe	4.32	0.99	< 0.001
	Putamen	3.49	0.80	0.006
	VIM/CM	2.95	0.68	0.030

Note: Group comparisons were performed using a weighted general linear model controlling for age and sex. Significance was determined at a connection-level *P* < 0.05 and cluster-level false discovery rate (FDR)-corrected *P* < 0.05. Effect sizes are reported as Hedges' g to provide unbiased estimates and correct for small-sample bias. GPi, globus pallidus internus. GPe, globus pallidus externus. STN, subthalamic nucleus. VIM/CM, ventral intermediate nucleus/centromedian nucleus of the thalamus.

Initially, local intra-regional hyperconnectivity within M1 was observed between the composite inter-effector and effector-specific regions (*t* = 2.71, P_FDR_ = 0.020, Hedges' g = 0.62). Furthermore, we identified extensive subsystem-specific hyperconnectivity between M1 subregions and the six a priori subcortical nuclei. Specifically, the inter-effector region exhibited significantly increased FC with core basal ganglia nodes, including the GPi, STN, and GPe (all P_FDR_ < 0.05, Hedges' g ranging from 0.58 to 0.94). Notably, the effector-specific region demonstrated a more widespread pattern of hyperconnectivity, encompassing not only the aforementioned basal ganglia regions but also extending to the putamen and the thalamic VIM/CM nuclei (all P_FDR_ < 0.05, Hedges' g ranging from 0.68 to 1.09). These distinct connectivity profiles provide direct quantitative evidence of pathological network dedifferentiation, wherein the typical selective boundaries of cortico-subcortical circuits are compromised in PD.

### 3.3 Seed-to-voxel and network segregation analyses

The seed-to-voxel analysis revealed extensive and distinct alterations with large effect sizes (peak Hedges' g ranging from 0.95 to 1.36) in overall brain FC among patients with PD compared with HCs, with particularly divergent profiles observed between the inter-effector and effector-specific seed regions. Specifically, the primary effect of group showed that PD patients exhibited reduced connectivity primarily within the sensorimotor network, including the bilateral precentral and postcentral gyri, the supplementary motor area, and the Rolandic operculum, as well as in the right insula and right medial temporal structures (e.g., the hippocampus and parahippocampal gyrus). Conversely, increased connectivity was identified in the cerebellum (bilateral lobules VI, Crus I, and vermis), the right inferior parietal lobule, and bilateral prefrontal regions (superior and middle frontal gyri). To formally evaluate the shared and divergent network pathologies, a spatial conjunction analysis was conducted, followed by a direct statistical contrast and detailed assessments of subregion-specific alterations.

**Supplementary Fig. S2:**
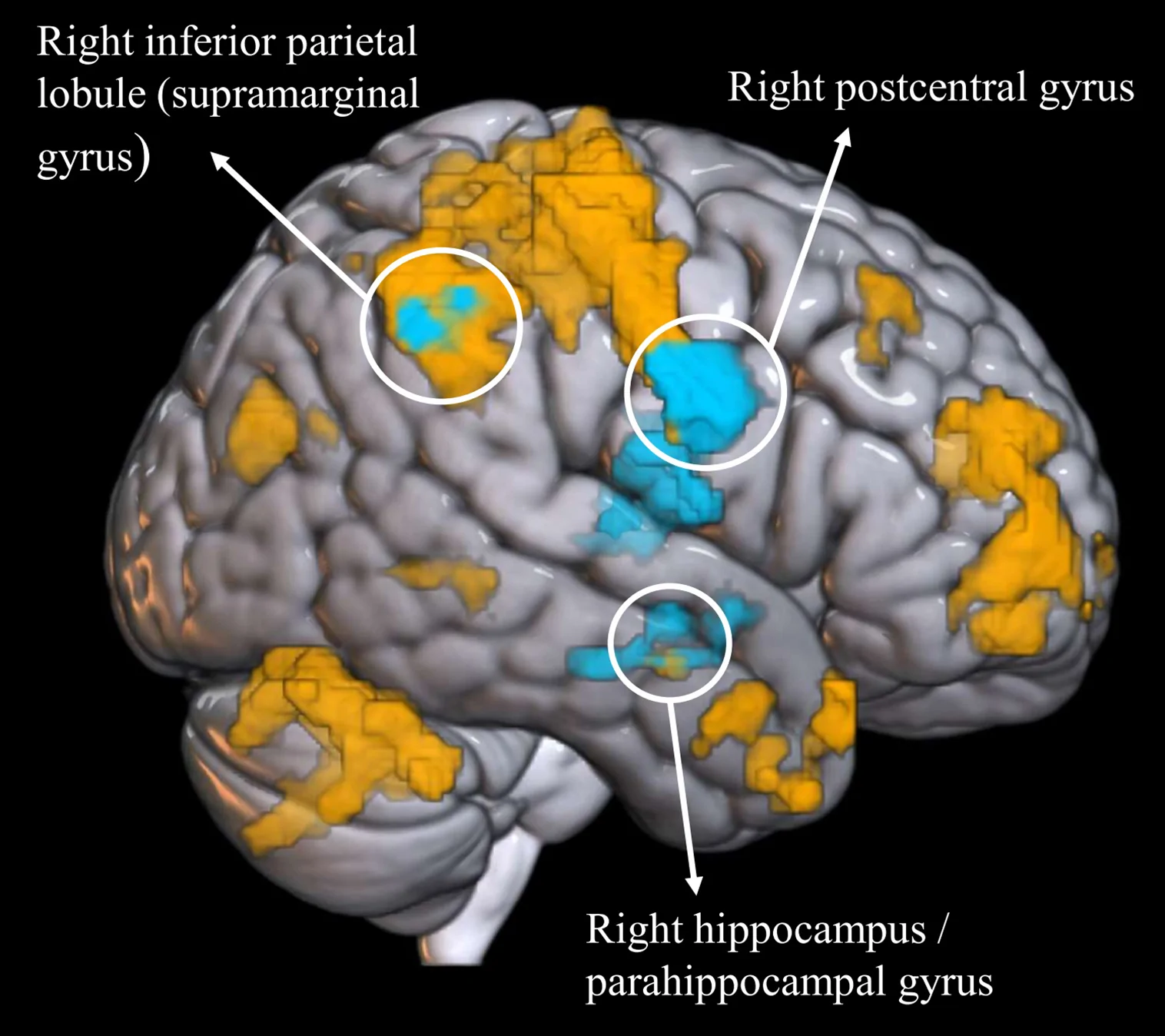
Spatial overlap and divergence of subregion-specific primary motor cortex (M1) functional connectivity alterations in Parkinson’s disease (PD). Three-dimensional brain surface rendering illustrates the altered whole-brain functional connectivity networks (Parkinson’s Disease vs. Healthy Control) seeded from the inter-effector region (blue) and the effector-specific region (yellow). The spatial conjunction (overlap) of connectivity alterations from both M1 subregions is highlighted by white circles. These shared network alterations are localized exclusively to the right hemisphere, encompassing the right inferior parietal lobule/supramarginal gyrus (cluster size = 156 voxels), right postcentral gyrus (cluster size = 114 voxels), and right medial temporal structures, including the hippocampus and parahippocampal gyrus (cluster size = 50 voxels). Notably, quantitative evaluation using the Dice similarity coefficient (DSC = 0.0735) confirms a high degree of spatial divergence: over 92% of the significantly altered network topologies are non-overlapping, underscoring the highly subsystem-specific nature of these network reorganizations.

Spatial Conjunction and Shared Pathological Core. A conjunction analysis formally evaluated the spatial overlap between the abnormal networks seeded from the two M1 subregions. The Dice similarity coefficient (DSC) between the two resulting statistical masks was 0.0735. As a spatial metric ranging from 0 (completely disjoint networks) to 1 (perfect overlap), this low DSC value quantitatively confirms that over 92% of the significantly altered network topologies are non-overlapping, underscoring that the two M1 subregions underwent highly divergent spatial reorganizations. Despite this profound divergence, a specific pattern of shared network alterations emerged, localized exclusively to the right hemisphere (Supplementary Fig. 2). Both subregions exhibited reduced positive FC with the right postcentral gyrus and right medial temporal structures (hippocampus and parahippocampal gyrus), alongside a concurrent decrease in negative FC (i.e., a loss of typical anti-correlation) with the right inferior parietal lobule (supramarginal gyrus).

**Supplementary Fig. S3:**
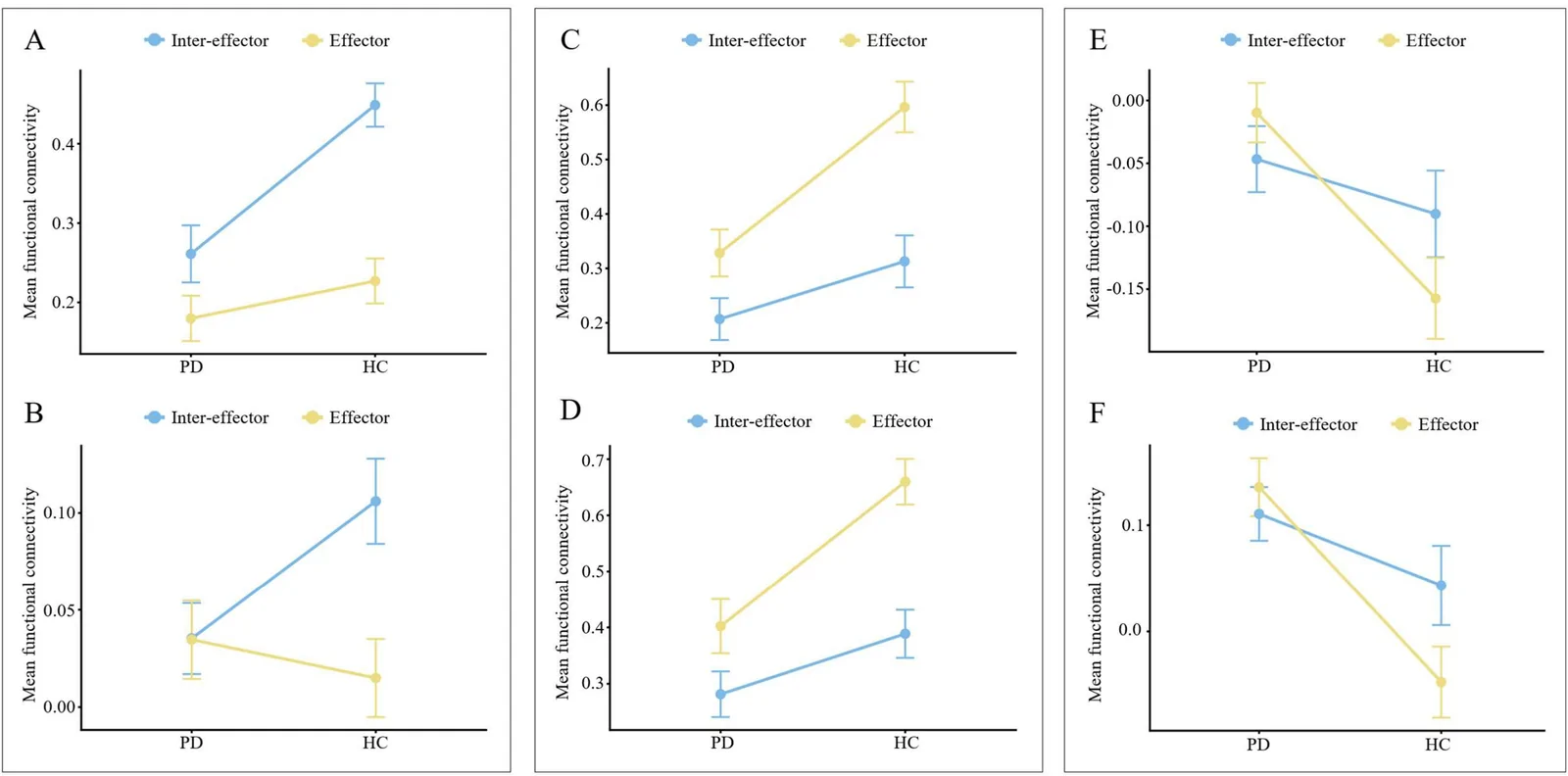
Distinct patterns of subregion-specific network reorganizations derived from the Group × Seed interaction analysis. Line plots illustrate the mean functional connectivity (Fisher’s Z-transformed values) extracted from six representative clusters exhibiting highly significant Group (Parkinson’s Disease vs. Healthy Control) × Seed (Inter-effector vs. Effector-specific) interaction effects (P < 0.001, cluster-level P_FDR_ < 0.05. Blue and yellow lines represent connectivity seeded from the inter-effector and effector-specific regions, respectively. Post-hoc evaluations of these interaction clusters revealed three divergent pathological patterns: (A, B) An “inter-effector-specific” disconnection in the left Rolandic operculum/superior temporal gyrus (A) and right putamen (B), where connectivity deficits in these auditory-somatosensory and basal ganglia hubs were exclusively driven by the inter-effector seed. (C, D) A profound sensorimotor disconnection in the right postcentral/precentral gyrus (C) and bilateral paracentral lobule/supplementary motor area (D). Although both seeds exhibited reduced connectivity in the PD group, the effector-specific seed showed a significantly more pronounced decoupling. (E, F) A “pathological desegregation” pattern strictly confined to the effector-specific seed in the left cerebellar Crus II (E) and right cerebellar lobule VI (F). In these regions, the effector-specific seed exhibited a dramatic transition from typical anticorrelation (negative connectivity in the HC group) to aberrant positive connectivity (in the PD group). Error bars indicate standard errors.

Direct Statistical Contrast and Subregion Dissociation. While the conjunction analysis identified a spatially restricted shared pathological core, the whole-brain 2 × 2 mixed ANCOVA revealed highly significant Group × Seed interaction effects across widespread distributed networks (Supplementary Table 2; Supplementary Fig. 3), statistically corroborating the profound subregion dissociation. Post-hoc evaluations of these interaction clusters revealed three distinct divergent patterns. First, an “inter-effector specific” disconnection was observed, where connectivity deficits with auditory-somatosensory integration hubs (e.g., bilateral Rolandic operculum and superior temporal gyrus) and core basal ganglia nodes (putamen) were exclusively driven by the inter-effector seed. Second, a profound sensorimotor disconnection emerged: although both seeds exhibited reduced connectivity with the core sensorimotor cortices (precentral/postcentral gyri and paracentral lobule), the effector-specific seed showed a significantly more severe decoupling. Third, a “pathological desegregation” pattern was strictly confined to the effector-specific seed, characterized by a dramatic transition from typical anti-correlation to aberrant positive connectivity with bilateral cerebellar regions (Crus II, Lobule VI).

**Fig. 1:**
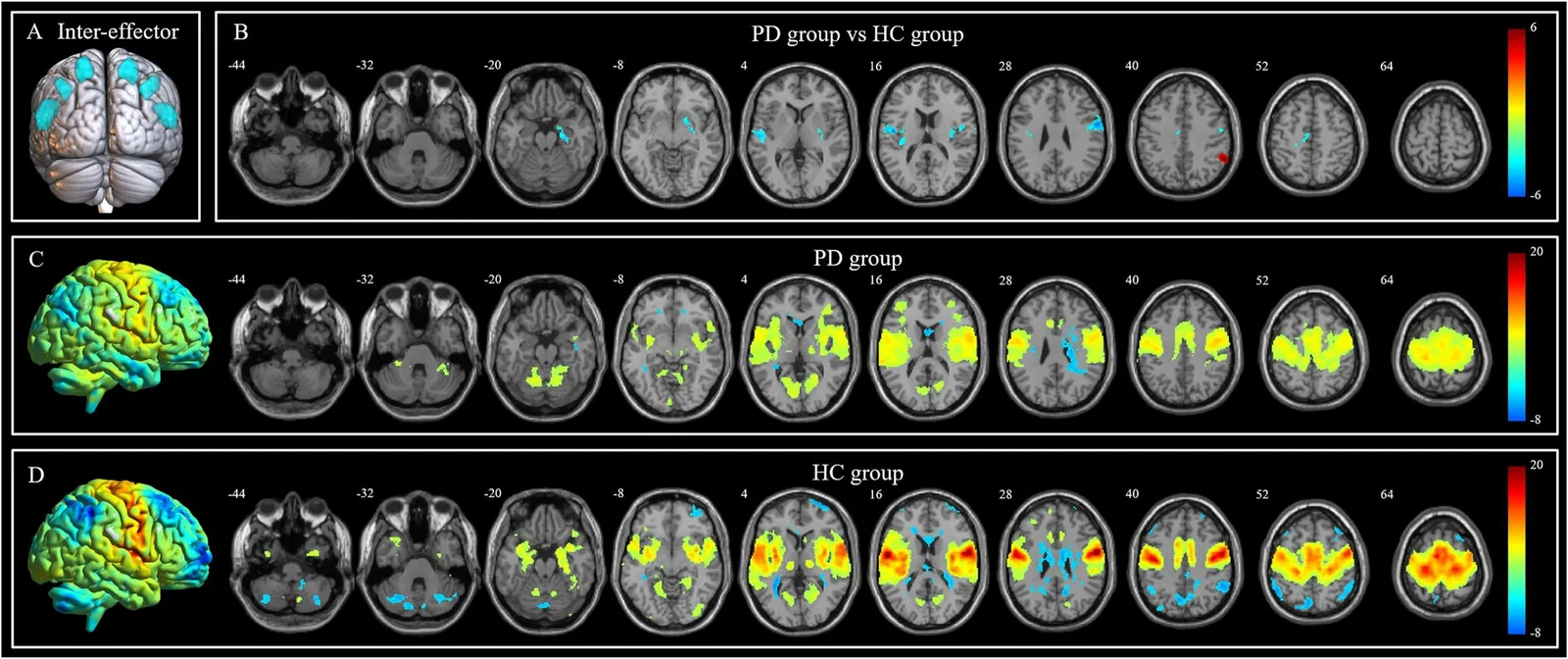
Whole-brain functional connectivity (FC) of the primary motor cortex (M1) inter-effector region. (A) The M1 inter-effector region serves as a seed region. (B) Differences between groups (Parkinson's disease (PD) vs. healthy control (HC)) reveal disease-related changes in FC, with warm colors indicating PD > HC (hyperconnectivity) and cool colors indicating PD < HC (hypoconnectivity). (C) and (D) show the positive (warm colors) and negative (cool colors) FC patterns for the PD and HC groups, respectively. All maps are thresholded at a voxel-level *P* < 0.001 and a cluster-level *P* < 0.05 (FDR corrected).

Subregion-Specific Dysconnectivity Profiles. Beyond the shared core pathology, the two subregions demonstrated highly distinct patterns of dysconnectivity. For the inter-effector seed, PD patients showed a selective profile of decreased positive FC with several regions (Table 3, Fig. 1): (1) bilateral sensorimotor cortices (precentral and postcentral gyri); (2) auditory and somatosensory integration areas, including the bilateral Rolandic operculum, left superior temporal gyrus, and Heschl's gyrus; and (3) bilateral insula.

**Table 3:** Alterations in primary motor cortex (M1) Inter-Effector Region Functional Connectivity with the Whole-Brain: Parkinson's Disease vs. Healthy Control.

Brain regions	Cluster size	MNI coordinates			T-value (at peak)	Hedges' g	P_FDR_ (Cluster-level)
		X	Y	Z			
Cluster 1 (positive FC decrease)	**409**	**60**	**2**	**30**	**−5.585**	**1.28**	**< 0.001**
Right postcentral gyrus	222	60	−4	32			
Right precentral gyrus	169	58	2	30			
Cluster 2 (positive FC decrease)	**386**	**−48**	**−8**	**16**	**−5.216**	**1.20**	**< 0.001**
Left superior temporal gyrus	106	−54	−14	6			
Left rolandic operculum	101	−52	−4	12			
Left postcentral gyrus	77	−54	−6	18			
Left Heschl's gyrus	23	−58	−12	8			
Left precentral gyrus	19	−52	−2	20			
Left insula	7	−36	−8	18			
Cluster 3 (positive FC decrease)	**184**	**20**	**−12**	**−16**	**−4.505**	**1.04**	**< 0.001**
Right hippocampus	63	24	−12	−16			
Right amygdala	39	26	−2	−14			
Right parahippocampal gyrus	25	24	−18	−18			
Right putamen	10	20	8	−10			
Cluster 4 (positive FC decrease)	**181**	**44**	**−10**	**20**	**−4.667**	**1.07**	**< 0.001**
Right rolandic operculum	75	46	−6	18			
Right insula	54	34	−8	14			
Right putamen	19	32	−12	8			
Cluster 5 (negative FC decrease)	**157**	**58**	**−50**	**42**	**5.022**	**1.16**	**0.002**
Right inferior parietal lobule	114	56	−48	42			
Right supramarginal gyrus	42	56	−44	42			

Note: FC, functional connectivity. Positive FC decrease: the HC group shows positive FC, while the PD group shows weaker positive FC. Negative FC decrease: the HC group shows negative FC, while the PD group shows weaker negative FC.

**Fig. 2:**
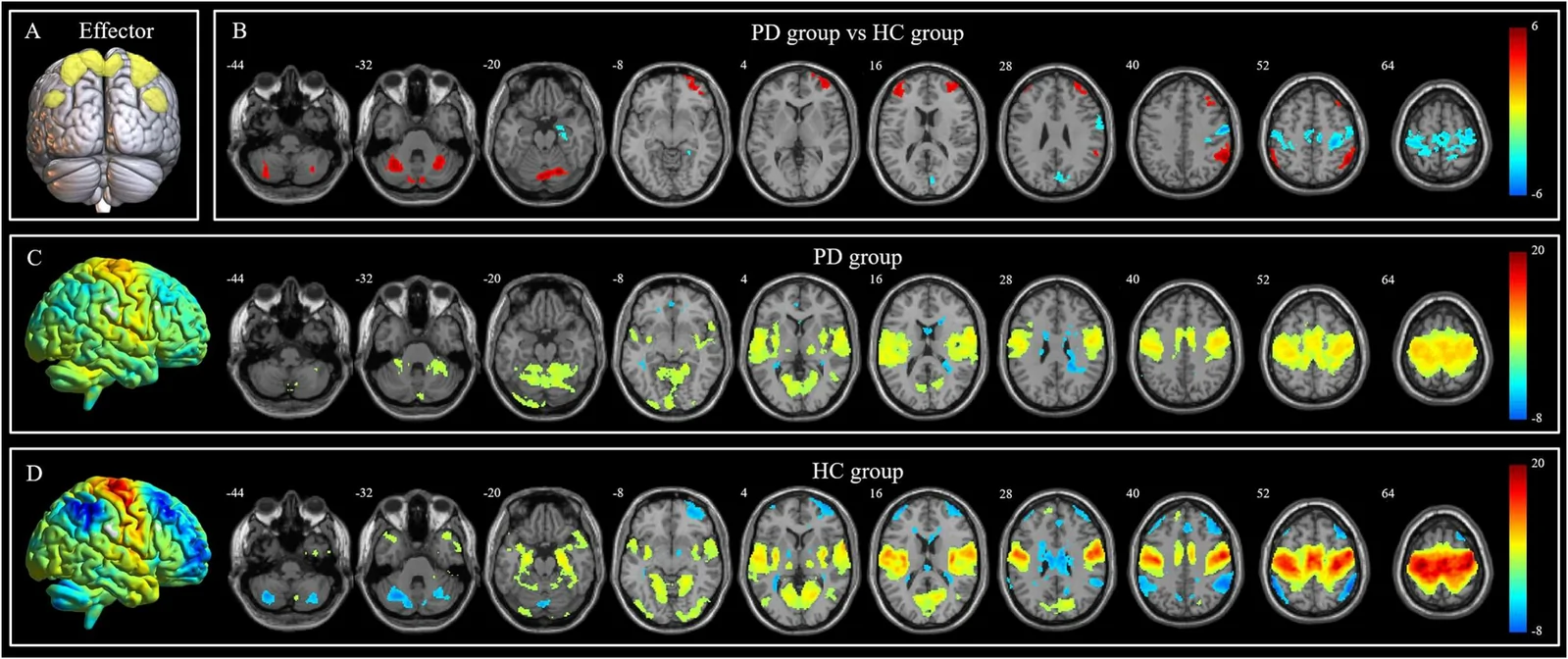
Whole-brain functional connectivity (FC) of the primary motor cortex (M1) effector region. (A) The M1 effector region serves as a seed region. (B) Differences between groups (Parkinson's disease (PD) vs. healthy control (HC)) show disease-related changes in FC, with warm colors indicating PD > HC (hyperconnectivity) and cool colors indicating PD < HC (hypoconnectivity). (C) and (D) display positive (warm colors) and negative (cool colors) FC patterns for the PD and HC groups, respectively. All maps are thresholded at voxel-level *P* < 0.001 and cluster-level *P* < 0.05 (FDR corrected).

For the effector-specific seed, PD patients exhibited significantly greater reductions in FC (Table 4, Fig. 2). Decreased positive FC was observed with: (1) the core sensorimotor network (bilateral precentral/postcentral gyri, supplementary motor area, paracentral lobule, and superior parietal lobule); and (2) right occipito-parietal regions (precuneus, cuneus, and calcarine cortex). Furthermore, patients with PD exhibited significantly decreased negative FC (i.e., loss of anti-correlation) between the effector-specific seed and an extensive array of regions. These encompassed broad cerebellar areas, including bilateral lobules VI, VIIb, VIII, Crus I/II, and vermis VI/VII, as well as regions within the frontoparietal cognitive control network, such as the bilateral superior, middle, and inferior frontal gyri, and bilateral inferior parietal lobules.

**Table 4:** Alterations in primary motor cortex (M1) Effector Region Functional Connectivity with the Whole-Brain: Parkinson's Disease vs. Healthy Control.

Brain regions	Cluster size	MNI coordinates			T-value (at peak)	Hedges' g	P_FDR_ (Cluster-level)
		X	Y	Z			
Cluster 1 (positive FC decrease)	**3577**	**−34**	**−26**	**72**	**−5.930**	**1.36**	**< 0.001**
Right precentral gyrus	776	38	−18	60			
Right postcentral gyrus	726	42	−26	52			
Left postcentral gyrus	598	−36	−30	58			
Left paracentral lobule	374	−8	−26	66			
Left precentral gyrus	308	−34	−22	66			
Right supplementary motor area	166	8	−20	62			
Right paracentral lobule	81	6	−30	64			
Left supplementary motor area	71	−4	−16	54			
Left precuneus	56	−12	−42	68			
Left superior parietal lobule	44	−24	−44	62			
Right superior parietal lobule	34	18	−48	62			
Right middle frontal gyrus	29	52	−8	54			
Left middle cingulate gyrus	15	−4	−8	48			
Right superior frontal gyrus	10	28	−10	64			
Right precuneus	6	12	−48	66			
Cluster 2 (negative FC decrease)	**1719**	**34**	**−50**	**−36**	**5.447**	**1.25**	**< 0.001**
Right cerebellum/lobule VI	495	26	−64	−26			
Left cerebellum/lobule VI	306	−26	−62	−28			
Left cerebellum/Crus I	296	−28	−66	−30			
Left cerebellum/Crus II	197	−32	−72	−42			
Right cerebellum/Crus I	180	30	−62	−34			
Bilateral vermis/lobule VI	64	2	−72	−18			
Right cerebellum/Crus II	40	10	−76	−30			
Bilateral vermis/ lobule VII	30	2	−76	−22			
Left cerebellum/lobule VIIb	13	−32	−62	−44			
Right cerebellum/lobule VIIb	10	34	−62	−44			
Right cerebellum/lobule VIII	5	32	−64	−44			
Cluster 3 (negative FC decrease)	**775**	**28**	**56**	**−12**	**5.664**	**1.30**	**< 0.001**
Right middle frontal gyrus	472	36	54	6			
Right superior frontal gyrus	195	34	58	8			
Cluster 4 (negative FC decrease)	**732**	**52**	**−48**	**46**	**5.358**	**1.23**	**< 0.001**
Right inferior parietal lobule	449	52	−48	46			
Right supramarginal gyrus	250	58	−42	38			
Right angular gyrus	25	46	−58	52			
Cluster 5 (negative FC decrease)	**228**	**−38**	**52**	**26**	**4.461**	**1.03**	**< 0.001**
Left middle frontal gyrus	211	−40	50	18			
Left inferior frontal gyrus (pars triangularis)	14	−40	44	12			
Cluster 6 (positive FC decrease)	**181**	**8**	**−78**	**24**	**−4.763**	**1.10**	**< 0.001**
Right cuneus	154	8	−78	24			
Right precuneus	12	4	−68	24			
Right calcarine fissure and surrounding cortex	9	8	−80	16			
Cluster 7 (negative FC decrease)	**95**	**−52**	**−54**	**52**	**4.390**	**1.01**	**0.018**
Left inferior parietal lobule	67	−54	−48	50			
Cluster 8 (negative FC decrease)	**82**	**36**	**32**	**46**	**4.206**	**0.97**	**0.024**
Right middle frontal gyrus	77	36	32	44			
Cluster 9 (positive FC decrease)	**70**	**20**	**−4**	**−18**	**−4.255**	**0.98**	**0.039**
Right hippocampus	51	24	−12	−18			
Right parahippocampal gyrus	13	24	−16	−18			
Cluster 10 (positive FC decrease)	**67**	**26**	**−42**	**−2**	**−4.110**	**0.95**	**0.041**
Right parahippocampal gyrus	20	26	−42	−6			
Right fusiform gyrus	8	32	−50	−4			
Right hippocampus	6	28	−38	−2			

Note: FC, functional connectivity. Positive FC decrease: the HC group shows positive FC, while the PD group shows weaker positive or negative FC. Negative FC decrease: the HC group shows negative FC, while the PD group shows weaker negative or positive FC.

Quantitative Evaluation of Network Segregation. To provide a quantitative evaluation of these qualitative dysconnectivity patterns, we calculated the localized System Segregation indices for both M1 subregions relative to their respective structurally uncoupled targets. For the inter-effector region, its functional segregation from the right inferior parietal lobule (supramarginal gyrus) was significantly reduced in patients with PD compared to HCs (PD: 0.639 ± 0.637 vs. HC: 0.968 ± 0.072; *t* = −3.12, *P* = 0.003). More strikingly, for the effector-specific region, the loss of normal anti-correlation with external networks translated mathematically into a severe collapse of functional segregation. By setting normal negative correlations to zero per the established methodology to isolate the pathological intrusion of anomalous positive couplings, HCs exhibited near-perfect segregation from these networks (Segregation index ≈ 1.0). In contrast, PD patients showed a drastically reduced numerical segregation metric driven by the pathological emergence of anomalous positive connectivity, both in relation to the cerebellar network (lobules VI/VII and Crus I/II; PD: 0.449 ± 0.764 vs. HC: 0.927 ± 0.096; *t* = −3.77, *P* < 0.001) and the distributed frontoparietal network (PD: 0.616 ± 0.615 vs. HC: 0.941 ± 0.040; *t* = −3.21, *P* = 0.003). These explicit numeric indicators quantitatively substantiate the breakdown of large-scale network boundaries in PD.

### 3.4 Correlation analyses

**Fig. 3:**
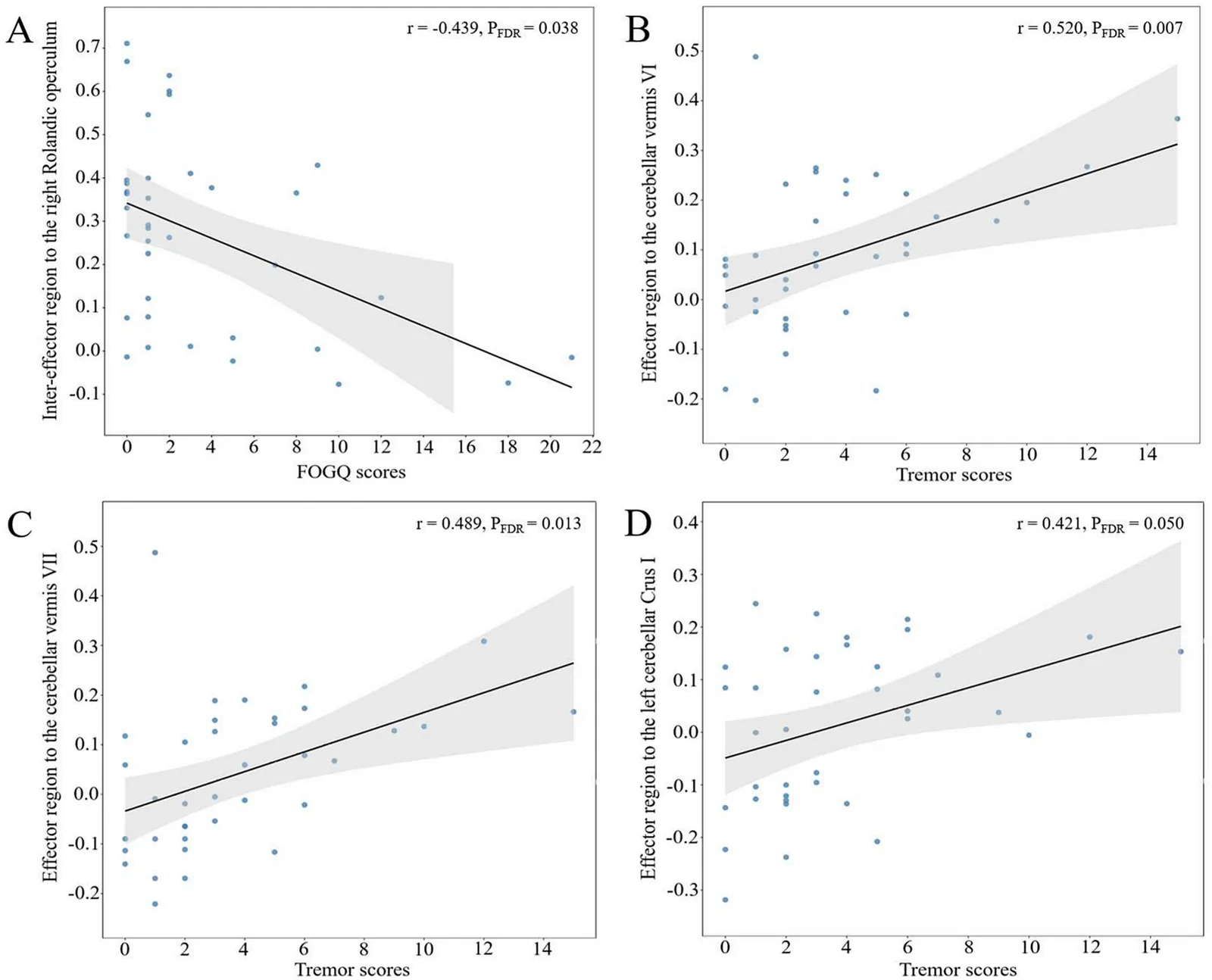
Subregion-specific functional connectivity (FC) correlates with distinct motor symptoms in patients with PD. (A) Scatter plot illustrating the significant partial correlation between the FC of the inter-effector region to the right Rolandic operculum and the Freezing of Gait Questionnaire (FOGQ) scores. (B—D) Scatter plots demonstrating the significant partial correlations between the aberrant FC of the effector-specific region to distinct cerebellum subregions and Tremor Scores: (B) Cerebellar Vermis VI, (C) Cerebellar Vermis VII, and (D) left Crus I. For all panels, the solid lines represent the linear regression fits, and the shaded areas indicate the 95% confidence intervals. The displayed correlation coefficients (r) and *P*-values are derived from partial correlation analyses strictly controlling for age, sex, and levodopa equivalent daily dose (LEDD).

Partial correlation analyses, controlling for age, sex, and LEDD, revealed significant associations between region-specific FC and clinical scores (FDR-corrected *P* < 0.05; Fig. 3).

Hypoconnectivity between the inter-effector region and the right Rolandic operculum was significantly associated with greater severity of freezing of gait (FOGQ Scores: *r* = −0.439, P_FDR_ = 0.038).

For the effector region, connectivity with the cerebellar vermis showed strong positive correlations with tremor severity. Specifically, FC with vermis VI (*r* = 0.520, P_FDR_ = 0.007) and vermis VII (*r* = 0.489, P_FDR_ = 0.013) were both significantly associated with higher Tremor Scores. A trend-level positive association was also observed between effector region connectivity to the left cerebellar Crus I and Tremor Scores (*r* = 0.421, P_FDR_ = 0.050).

## 4 Discussion

Using a subregion-specific fMRI approach and rigorous statistical validation, this study delineated a multilayered network pathology within the M1 in PD. By segmenting M1 into inter-effector and effector-specific subregions, we identified four core patterns of reorganization: (1) Local and cortico-subcortical hyperconnectivity, which may reflect a “dual pathology” of intra-cortical dedifferentiation and compromised subcortical boundaries; (2) Subsystem-specific long-range dysconnectivity, characterized by divergent hypoconnectivity profiles across sensorimotor and associative networks; (3) Quantitatively verified impaired network segregation, evidenced by a severe collapse in the System Segregation index and a pathological loss of anti-correlations with cerebellar and frontoparietal networks; and (4) Symptom-specific clinical correlations, linking inter-effector hypoconnectivity to freezing of gait, and aberrant effector-specific-cerebellar positive coupling to tremor severity.

### 4.1 Altered functional connectivity profiles of M1 subregions

Our expanded ROI-to-ROI analysis revealed a broad pattern of pathological hyperconnectivity within the cortico-basal ganglia-thalamic network, characterized by a distinct subsystem-specific topography. Notably, the inter-effector region exhibited a significant increase in FC with core basal ganglia nodes, including the GPi, STN, and GPe. This finding provides critical in vivo validation of the emerging framework proposed by Ren et al. (2026), which fundamentally reconceptualizes PD as a “SCAN disorder”. According to this model, dopaminergic depletion leads to a pathological overdrive from basal ganglia loops that is preferentially directed toward cortical SCAN nodes. Our observation of robust STN/GPi/GPe-to-inter-effector hyperconnectivity strongly corroborates this premise, which may reflect a core pathophysiological disruption that could impair the SCAN's capacity to integrate whole-body action planning with physiological arousal.

Interestingly, while recent models have emphasized selective SCAN vulnerability (Ren et al., 2026), our results extend this framework by revealing an even more widespread hyperconnectivity profile within the effector-specific region. Beyond its hyperconnectivity with the GPi, STN, and GPe—which could reflect a global, network-wide loss of basal ganglia inhibitory control over the motor cortex due to progressive dopaminergic denervation (Baudrexel et al., 2011)—the effector-specific region uniquely demonstrated pathological coupling with the putamen and the thalamic VIM/CM nuclei. The putamen and VIM/CM are classical hubs of the primary sensorimotor loops, strongly implicated in motor execution and tremor genesis, respectively. Elevated resting-state coupling between the putamen and primary motor cortices is a recognized hallmark of altered cortico-striatal dynamics in PD. This hyperconnectivity has often been tentatively interpreted either as a maladaptive loss of functional segregation within the motor network or as a compensatory cortico-striatal reorganization aimed at maintaining motor output despite severe dopamine depletion (Kwak et al., 2010). Concurrently, the aberrant coupling between the VIM and the effector-specific region underscores the cerebello-thalamo-cortical circuit's involvement in classical resting tremor (Helmich et al., 2012). Collectively, our data suggest a “dual pathology”: while the SCAN is compromised by basal ganglia overdrive that affects action-arousal integration, the effector system is further burdened by widespread dysfunction across classical sensorimotor and tremor-generating loops.

Beyond these cortico-subcortical disruptions, we observed a significant increase in local FC between the inter-effector and effector-specific regions within M1. This finding aligns with the contemporary “integrate-isolate” model, which posits that the inter-effector region (as part of the SCAN) coordinates whole-body actions, whereas the effector-specific region facilitates isolated, fine motor control (Gordon et al., 2023). In the context of the aforementioned subcortical-to-SCAN overdrive (Ren et al., 2026), this intra-M1 hyperconnectivity has traditionally been speculated to represent a potentially compensatory cortical adaptation to impaired basal ganglia output (McGregor and Nelson, 2019). Under this framework, as automated motor execution diminishes, the hyperactive SCAN engages effector-specific subregions to sustain voluntary movement through effortful, consciously regulated control. However, an equally plausible alternative is that this hyperconnectivity could reflect a maladaptive pathogenic process. Alternatively, it may reflect pathological dedifferentiation—a collapse of the highly specialized functional boundaries separating these M1 subregions—or a progressive loss of local intracortical surround inhibition secondary to dopaminergic denervation. Under this “hyperconnectivity paradox”, the aberrant, generalized synchronization across M1 networks could inherently contribute to movement rigidity and motor inefficiency, rather than serving a purely protective role.

### 4.2 Whole-brain dysconnectivity of M1 inter-effector region

Our findings reveal that the inter-effector region, which integrates whole-body action planning with physiological and cognitive control, undergoes selective functional disconnection in PD. This region shows reduced positive FC with nodes governing primary sensorimotor execution (precentral/postcentral gyri) (Baudrexel et al., 2011), auditory-motor articulation (superior temporal gyrus, Rolandic operculum) (Chen and Watson, 2017), and emotional-mnemonic processing (hippocampus, amygdala, insula) (Carey et al., 2021). This specific topography of dysconnectivity aligns with the recent characterization of the SCAN as a hub for coordinating whole-body action with arousal and physiological states (Ren et al., 2026), suggesting a disintegration of the high-order SCAN, compromising the brain's capacity for seamless sensorimotor integration and for embedding cognitive-emotional states into action plans (Gordon et al., 2023). This network-level failure provides a plausible neural substrate for core axial motor deficits (e.g., postural instability, gait freezing) and prevalent non-motor symptoms in PD (Ryman and Poston, 2020).

Furthermore, we observed reduced negative FC between the inter-effector region and the right inferior parietal lobule (supramarginal gyrus). In a healthy brain, this anti-correlation likely reflects functional segregation, in which the SCAN is decoupled from the ventral attention network, isolating action execution from distracting sensory signals—a process potentially controlled by networks such as the Action-Mode Network (Dosenbach et al., 2025). In PD, the weakening of this negative coupling suggests a pathological blurring of network boundaries (Fox et al., 2005), a phenomenon robustly corroborated by the significantly diminished System Segregation index observed in our quantitative analysis (Chan et al., 2014). This loss of segregation might allow abnormal cross-talk, potentially disrupting focused motor execution by integrating irrelevant external sensory and attentional information, thereby linking motor deficits with cognitive symptoms (Gordon et al., 2023; Tessitore et al., 2019).

### 4.3 Whole-brain dysconnectivity of M1 effector-specific region

We observed extensive functional decoupling between the effector-specific region and distributed brain areas, underscoring the profound network disruption inherent to PD (Baudrexel et al., 2011). First, hypoconnectivity with primary sensorimotor cortices, the supplementary motor area, and the superior parietal lobule suggests a possible disintegration of typical motor-execution circuits, which may contribute to bradykinesia and reduced movement precision (Wu et al., 2011). Second, reduced coupling with occipito-parietal regions (precuneus, cuneus) could reflect impaired integration of visual feedback for online motor correction, which is highly relevant to postural instability (Oliveira et al., 2025). Third, diminished connectivity with medial temporal lobe structures links motor dysfunction to memory systems, aligning with the co-occurring navigational and memory deficits frequently observed in PD (Chen et al., 2024).

A particularly notable finding was the pathological shift from normal anti-correlation in HCs to aberrant positive connectivity in PD patients between the M1 effector-specific region and cerebellar lobules VI/VII and Crus I/II. This shift signifies a fundamental reorganization of cortico-cerebellar interactions, reflecting a loss of network specificity and possibly indicating maladaptive, pathological coupling. Such aberrant synchronization is more consistent with a dysfunctional correlate integral to the disease process itself than with a mere compensatory response (Li et al., 2023).

Furthermore, we found attenuated anti-correlations (reduced negative FC) between the effector-specific region and a distributed frontoparietal network encompassing the bilateral middle/superior/inferior frontal gyri and the inferior parietal lobules—core nodes of the cognitive control and ventral attention systems (Corbetta and Shulman, 2002; Vincent et al., 2008). In the healthy brain, anti-correlations between motor execution and these cognitive networks are crucial for isolating automatic movements from interfering cognitive processes (Dosenbach et al., 2007; Fox et al., 2005). The widespread attenuation of these anti-correlations in PD signifies a pathological collapse of this protective segregation (Baggio et al., 2015), potentially forcing patients to rely on effortful cognitive control for previously automatic movements (Dirkx et al., 2016). This consequence was mathematically validated through a severe, statistically significant collapse in the effector-specific region's System Segregation indices. This inefficient neural resource allocation may underlie both degraded motor skills and concomitant cognitive impairments (Gratton et al., 2018).

Finally, given the characteristic asymmetric motor onset of PD, we conducted a post hoc subgroup analysis to evaluate potential lateralization effects by comparing patients with left-sided (LPD, *n* = 17) versus right-sided (RPD, *n* = 14) symptom onset. No significant differences in whole-brain FC were observed between the LPD and RPD subgroups for either M1 subregion. This absence of lateralized FC differences suggests that the multilayered network alterations identified in our study reflect a systemic, global pathological reorganization of brain networks, rather than a localized secondary adaptation driven solely by unilateral motor symptoms. Furthermore, this finding methodologically justifies our primary analytical approach of evaluating the combined PD cohort using bilateral composite ROIs, as the relatively balanced distribution of symptom onset effectively mitigated lateralization bias, confirming that the observed FC alterations represent robust, core pathophysiological features of PD.

### 4.4 Statistical dissociation of M1 subregion pathologies

The direct statistical contrast provided by the Group × Seed interaction definitively substantiates our hypothesis of a “dual pathology” in PD, confirming that the visually distinct dysconnectivity profiles are driven by mathematically divergent network reorganizations. This statistical dissociation solidifies our findings across three critical neurobiological dimensions.

First, the highly significant interaction effects involving the Rolandic operculum and putamen confirm that the isolation of these regions is exclusively an inter-effector (SCAN) pathology. As the SCAN functions as a central hub integrating physiological arousal with whole-body action plans (Gordon et al., 2023), its selective uncoupling from the putamen aligns with the pathological basal ganglia overdrive directed toward SCAN nodes (Ren et al., 2026). Furthermore, its specific disconnection from the Rolandic operculum—a critical sensorimotor integration hub—statistically isolates the neural substrate underlying complex axial failures, such as freezing of gait, which may contribute to impaired fluid locomotion under high cognitive demand.

Second, while both subregions share a fundamental deficit in the primary sensorimotor execution network (e.g., postcentral gyrus, paracentral lobule), the interaction analysis reveals that the effector-specific region exhibits a significantly more severe degree of decoupling. This disproportionate collapse underscores that the failure of isolated fine motor execution (e.g., bradykinesia) is borne predominantly by the effector-specific system.

Finally, the interaction explicitly confirms that the pathological loss of large-scale network segregation is uniquely confined to the effector-specific subregion. The aberrant reversal from anti-correlation to positive functional coupling with the cerebellar Crus II and Lobule VI is shown to be highly effector-specific. This exclusively aberrant synchronization may transform the cerebellum's customary inhibitory influence into a possible destabilizing influence that could promote oscillatory activity within thalamocortical networks (Zhong et al., 2022). By statistically isolating this maladaptive cortico-cerebellar coupling to the effector-specific network, our results provide robust, specific neural evidence for the generation and manifestation of tremor, cementing the effector-specific cerebellar circuit as a vital non-dopaminergic target for precision neuromodulation.

### 4.5 Clinical correlates of M1 subregional connectivity

Our correlation analyses demonstrate that distinct FC patterns within M1 subregions are associated with specific clinical symptoms, validating their pathophysiological relevance. Crucially, post-hoc partial correlation analyses controlling for age, sex, and LEDD revealed that these FC alterations did not correlate with disease duration or global motor severity (UPDRS-III total score). This independence from the global disease burden suggests that these network reorganizations represent a relatively stable pathophysiological trait that drives targeted clinical phenotypes, rather than merely reflecting a progressive state of generalized disease. Furthermore, the lack of correlation with the global UPDRS-III score underscores the robust symptom-specificity of our findings, reinforcing that these M1 subcircuits serve as precise neural substrates for specific symptoms rather than mere reflections of overall motor deterioration.

The degree of hypoconnectivity between the inter-effector region and the right Rolandic operculum was positively correlated with the severity of freezing of gait. This disconnection from a key sensorimotor integration hub could impair the precise sensory-motor processing required for fluid locomotion, predisposing patients to freezing episodes under high processing demand (Snijders et al., 2011). Notably, we did not find significant associations between inter-effector connectivity and cognitive/affective scores in this cohort, possibly due to the relatively small sample size and the early disease stage of our participants. We posit that inter-effector dysconnectivity may become more closely linked to cognitive-emotional symptoms in advanced PD.

Regarding the effector-specific region, the observed positive correlation between its aberrant FC with the cerebellar vermis and tremor severity is particularly noteworthy. This finding directly associates the cerebello-thalamo-cortical circuit with the pathophysiology of PD tremor (Helmich et al., 2012). Notably, this positive correlation reflects a pathological shift from typical anti-correlation to aberrant positive connectivity, indicating that abnormal synchronization within this circuit is not merely incidental but is closely associated with tremor pathophysiology. The underlying pathology, including α-synuclein accumulation and synaptic dysfunction in the cerebellum, may transform into a pattern consistent with a pro-oscillatory, destabilizing effect on thalamocortical networks (Zhong et al., 2022). Consequently, the loss of cerebellar inhibition—or its shift toward detrimental facilitation—could exacerbate tremor amplitude, a notion supported by evidence linking dentate nucleus activity to tremor manifestation (Ma et al., 2015). This cerebellum-centric mechanism offers a potential explanatory framework for the frequent resistance of tremor to dopaminergic therapy and underscores the cerebellum as a vital non-dopaminergic therapeutic target (Zhong et al., 2022).

### 4.6 Methodological and clinical implications

Our work underscores the necessity of a subregion-specific approach to investigating M1 in neurodegeneration. The distinct connectivity signatures we identified align with prior evidence that levodopa differentially modulates FC across M1 subregions, with specific patterns correlating with improvements in rigidity, bradykinesia, and tremor (Shen et al., 2020). By moving beyond a homogeneous view of M1, our results reinforce its functional heterogeneity and highlight its potential as a target for symptom-specific neuromodulation. These findings could inform next-generation neuromodulatory protocols, such as repetitive transcranial magnetic stimulation (rTMS). Precision targeting of symptom-specific M1 subregions, rather than treating M1 as a unitary structure, may enhance therapeutic efficacy for both motor and non-motor symptoms, supporting a precision medicine framework for PD (Zhang et al., 2022). Crucially, a recent randomized controlled trial has validated this strategy, demonstrating that rTMS targeting personalized SCAN nodes significantly accelerated symptom alleviation and yielded a twofold increase in clinical efficacy compared with stimulation of traditional effector-specific regions (Ren et al., 2026).

### 4.7 Limitations and future directions

Several limitations of this study must be acknowledged. First, the cross-sectional design precludes causal inferences about how FC changes with disease progression; consequently, it remains impossible to definitively determine whether the observed local M1 hyperconnectivity constitutes an adaptive compensatory response or a maladaptive pathophysiological consequence (e.g., pathological dedifferentiation, as conceptualized in the “hyperconnectivity paradox”). Longitudinal tracking of network evolution is therefore necessary. Second, scanning patients in the medicated “ON” state introduces a fundamental confound. As levodopa modulates M1 FC in a subregion-specific manner, the observed connectivity patterns inevitably reflect a compound of intrinsic disease pathology and pharmacological effects, which cannot be explicitly disentangled in the current cross-sectional design. Although we covaried for LEDD in our correlational analyses, this does not resolve the fundamental medication confound in the group comparisons. Future longitudinal studies incorporating drug-naive or OFF-state assessments are essential to isolate pure disease-driven signatures. Third, while our sample size (*n* = 37 per group) is adequate for typical clinical case-control comparisons, it remains modest for whole-brain seed-to-voxel inference. Consequently, the large effect sizes (Hedges' g = 0.95–1.36) reported herein, derived from peak voxels of significant clusters, are inherently susceptible to overestimation (a phenomenon often referred to as the “winner's curse” in neuroimaging). These values represent upper-bound estimates, and future large-scale, multisite cohorts are required to establish the exact, and likely more moderate, population-level effect sizes. Fourth, the relatively short rs-fMRI acquisition duration, while adequate for group-level comparisons, limits the reliability of individual-level estimates; longer scan durations would enhance test-retest stability and improve the detection of subject-specific network topologies. Fifth, the standard fMRI resolution limits the exploration of laminar or columnar organization within M1; advanced techniques, such as high-resolution layer-fMRI, could provide more detailed insights into these microcircuitries (Nothnagel et al., 2025). Sixth, we deliberately omitted global signal regression (GSR) to avoid artificially inducing negative correlations, relying instead on aCompCor. However, because preprocessing choices profoundly affect anti-correlation structures (Fox et al., 2009), the observed pathological loss of negative FC (network desegregation) must be interpreted cautiously in the context of our specific denoising pipeline. Finally, correlating FC with objective behavioral or physiological measures, rather than relying solely on semi-quantitative clinical scores, would enhance functional validation in future research.

## 5 Conclusion

In conclusion, this study clearly delineates a functionally specialized, subregion-specific reorganization of the M1 in PD, which is characterized by a distinct multilayered network pathology. First, local intra-cortical hyperconnectivity and subsystem-specific cortico-subcortical hyperconnectivity strongly point to a pathological dedifferentiation and a potentially compensatory yet possibly maladaptive shift toward effortful, consciously regulated motor control. Second, long-range dysconnectivity reveals a profound subsystem-specific breakdown: the inter-effector SCAN node disconnects from integrative hubs that bridge sensorimotor and cognitive-emotional processing, while the effector-specific region undergoes severe decoupling from core motor-execution circuits. Most critically, a widespread loss of normal anti-correlation signifies a fundamental failure of large-scale network segregation. This is quantitatively supported by the pathological reversal of cortico-cerebellar interactions—shifting from inhibitory to aberrantly facilitatory—and by the mathematically confirmed collapse of protective boundaries between motor execution and frontoparietal cognitive-control networks.

Collectively, these alterations allow us to reframe PD as a systems-level disorder that may be critically characterized by the dual impairment of network integration and segregation. The distinct connectivity signatures of M1 subregions—linking SCAN disconnection to axial failures (freezing of gait) and effector-specific cerebellar maladaptive coupling to tremor severity—provide a potential novel anatomical framework for understanding phenotypic heterogeneity in PD. Ultimately, this refined subregional perspective not only offers substantial advances in our pathophysiological understanding but also points toward targeted, circuit-based neuromodulatory strategies, moving the field beyond a homogeneous view of M1 toward precision therapies. Importantly, while the cross-sectional design precludes definitive causal claims, the consistency and strength of the observed connectivity patterns lend strong credence to these interpretations and warrant future longitudinal and experimental validation.

The following are the supplementary data related to this article.

Supplementary material 4

## CRediT authorship contribution statement

**Huazhi Li:** Writing – review & editing, Visualization, Validation, Software, Methodology, Investigation, Formal analysis, Data curation. **Wei Chen:** Writing – review & editing, Supervision, Resources, Investigation, Conceptualization. **Yapeng Qi:** Writing – review & editing, Methodology, Formal analysis, Data curation. **Wenshuang Tang:** Writing – review & editing, Validation, Software, Investigation, Data curation. **Chao Zhang:** Writing – review & editing, Resources, Investigation. **Hao Fan:** Writing – review & editing, Visualization, Resources, Investigation. **Liang Shu:** Writing – review & editing, Resources, Data curation. **Lipeng Lin:** Writing – review & editing, Resources, Investigation. **Jian-Ren Liu:** Writing – review & editing, Supervision, Resources, Funding acquisition, Conceptualization. **Xiaoxia Du:** Writing – review & editing, Writing – original draft, Supervision, Project administration, Methodology, Investigation, Conceptualization.

## Ethics statement

This study was approved by the Institutional Review Boards of Shanghai Ninth People's Hospital, Shanghai Jiao Tong University School of Medicine (2016–44-T1), East China Normal University (HR062–2018), and Shanghai University of Sport (202772023RT097). All procedures complied with the Declaration of Helsinki, and written informed consent was obtained from all participants.

## Declaration of generative AI and AI-assisted technologies in the writing process

During the preparation and revision of this work, the authors used Grammarly to improve language and clarity. After using this tool, the authors reviewed and edited the content as needed and take full responsibility for the publication's content. No other aspects of the research (including data analysis, figure generation, or scientific conceptualization) were assisted by AI.

## Funding

This work was supported by the General Project of Humanities and Social Sciences Research funded by the 10.13039/501100002701Ministry of Education (grant No. 23YJA890004), the Shanghai Municipal Education Commission-Gaofeng Clinical Medicine Grant Support (grant number 20161422), and the Natural Science Foundation Project funded by the 10.13039/501100003399Shanghai Municipal Science and Technology Commission (grant number 22ZR1436900).

## Declaration of competing interest

The authors declare that they have no known competing financial interests or personal relationships that could have appeared to influence the work reported in this paper.

## Data Availability

Data will be made available on request.
