# Supplementary material for: Altered functional connectivity in inter-effector and effector-specific subregions of the primary motor cortex in Parkinson's disease
Source: Neuroimage Clin. 2026 Aug 26;51:104051. doi: 10.1016/j.nicl.2026.104051 (PMC13545379; doi:10.1016/j.nicl.2026.104051)
Supplement: Supplementary file 1 [file mmc4.docx]

**Supplementary materials**

**STable. 1.** Quantitative Spatial Concordance of the Inter-Effector Subregions between the Current Derived Parcellation and the Gordon et al. Model.

| Subregions | The present study  MNI coordinates (X, Y, Z) | | | Gordon et al. (2023) | | | Euclidean distance (mm) |
| --- | --- | --- | --- | --- | --- | --- | --- |
|  |  |  |  | MNI coordinates (X, Y, Z) | | |  |
| S-L | -18 | -28 | 58 | -19 | -34 | 59 | 6.16 |
| S-R | 20 | -26 | 58 | 20 | -31 | 58 | 5.00 |
| M-L | -36 | -14 | 44 | -38 | -18 | 44 | 4.47 |
| M-R | 38 | -10 | 42 | 40 | -15 | 43 | 5.48 |
| I-L | -56 | -2 | 18 | -54 | -3 | 14 | 4.58 |
| I-R | 58 | 2 | 18 | 56 | -1 | 16 | 4.12 |

Note: MNI coordinates denote the peak voxels of the identified inter-effector components. S, Superior; M, Middle; I, Inferior; L, Left; R, Right.

**STable. 2.** Brain Regions Exhibiting Significant Group × Seed Interaction Effects in Whole-Brain Functional Connectivity.

| Brain regions | Cluster size | MNI coordinates | | | T-value  (at peak) | Hedges' g | P_FDR_  (Cluster-level) |
| --- | --- | --- | --- | --- | --- | --- | --- |
|  |  | X | Y | Z |  |  |  |
| Cluster 1 | **534** | **38** | **-22** | **46** | **6.054** | **1.39** | **< 0.001** |
| Right postcentral gyrus | 370 | 40 | -28 | 54 |  |  |  |
| Right precentral gyrus | 122 | 42 | -18 | 52 |  |  |  |
| Cluster 2 | **396** | **-56** | **10** | **4** | **-5.079** | **1.17** | **< 0.001** |
| Left rolandic operculum | 116 | -58 | 0 | 6 |  |  |  |
| Left inferior frontal gyrus (pars triangularis) | 65 | -46 | 24 | 6 |  |  |  |
| Left superior temporal gyrus | 49 | -58 | -10 | 6 |  |  |  |
| Left postcentral gyrus | 39 | -56 | -6 | 16 |  |  |  |
| Left inferior frontal gyrus (pars opercularis) | 35 | -56 | 12 | 4 |  |  |  |
| Left temporal pole: superior temporal gyrus | 31 | -52 | 10 | -2 |  |  |  |
| Left Heschl’s gyrus | 28 | -56 | -12 | 8 |  |  |  |
| Cluster 3 | **388** | **-8** | **-24** | **72** | **4.967** | **1.14** | **< 0.001** |
| Left paracentral lobule | 168 | -6 | -28 | 68 |  |  |  |
| Right paracentral lobule | 141 | 6 | -32 | 66 |  |  |  |
| Right supplementary motor area | 43 | 6 | -20 | 66 |  |  |  |
| Right precentral gyrus | 11 | 12 | -30 | 72 |  |  |  |
| Cluster 4 | **205** | **18** | **6** | **-10** | **-4.715** | **1.08** | **< 0.001** |
| Right putamen | 107 | 22 | 10 | -4 |  |  |  |
| Right caudate nucleus | 22 | 14 | 12 | 6 |  |  |  |
| Right pallidum | 15 | 20 | 6 | 2 |  |  |  |
| Cluster 5 | **180** | **54** | **-8** | **-2** | **-4.686** | **1.08** | **< 0.001** |
| Right superior temporal gyrus | 148 | 54 | -8 | 0 |  |  |  |
| Right rolandic operculum | 16 | 54 | 0 | 8 |  |  |  |
| Cluster 6 | **165** | **0** | **14** | **48** | **-4.164** | **0.96** | **0.001** |
| Left supplementary motor area | 94 | -2 | 18 | 48 |  |  |  |
| Left middle cingulate gyrus | 37 | -8 | 18 | 36 |  |  |  |
| Left supracallosal anterior cingulate cortex | 9 | -8 | 22 | 28 |  |  |  |
| Right supplementary motor area | 8 | 4 | 16 | 48 |  |  |  |
| Cluster 7 | **154** | **-26** | **-18** | **64** | **4.865** | **1.12** | **0.001** |
| Left precentral gyrus | 147 | -32 | -18 | 66 |  |  |  |
| Left postcentral gyrus | 5 | -30 | -30 | 66 |  |  |  |
| Cluster 8 | **119** | **-26** | **-74** | **-44** | **-4.576** | **1.05** | **0.005** |
| Left cerebellum/Crus II | 89 | -30 | -72 | -44 |  |  |  |
| Left cerebellum/lobule VIIb | 21 | -24 | -72 | -46 |  |  |  |
| Left cerebellum/lobule VIII | 9 | -20 | -68 | -46 |  |  |  |
| Cluster 9 | **110** | **-18** | **6** | **4** | **-4.726** | **1.09** | **0.007** |
| Left putamen | 57 | -20 | 6 | 4 |  |  |  |
| Left caudate nucleus | 24 | -12 | 12 | 6 |  |  |  |
| Left pallidum | 15 | -16 | 6 | 2 |  |  |  |
| Cluster 10 | **94** | **40** | **22** | **4** | **-4.859** | **1.12** | **0.012** |
| Right insula | 57 | 38 | 24 | 2 |  |  |  |
| Right inferior frontal gyrus (pars triangularis) | 18 | 40 | 24 | 6 |  |  |  |
| Cluster 11 | **94** | **36** | **-16** | **72** | **4.363** | **1.00** | **0.012** |
| Right precentral gyrus | 70 | 36 | -14 | 68 |  |  |  |
| Right superior frontal gyrus | 13 | 34 | -8 | 62 |  |  |  |
| Cluster 12 | **93** | **22** | **-56** | **64** | **4.295** | **0.99** | **0.012** |
| Right superior parietal lobule | 78 | 20 | -56 | 62 |  |  |  |
| Right postcentral gyrus | 6 | 24 | -46 | 58 |  |  |  |
| Cluster 13 | **90** | **18** | **-68** | **-18** | **-4.516** | **1.04** | **0.013** |
| Right cerebellum/lobule VI | 89 | 18 | -66 | -20 |  |  |  |
| Cluster 14 | **81** | **-58** | **-32** | **20** | **-4.319** | **0.99** | **0.018** |
| Left superior temporal gyrus | 68 | -60 | -34 | 20 |  |  |  |
| Left supramarginal gyrus | 8 | -62 | -38 | 24 |  |  |  |
| Cluster 15 | **67** | **44** | **-20** | **14** | **-4.838** | **1.11** | **0.036** |
| Right Heschl’s gyrus | 53 | 44 | -20 | 10 |  |  |  |
| Right rolandic operculum | 14 | 46 | -20 | 14 |  |  |  |
| Cluster 16 | **62** | **-26** | **-6** | **-8** | **-4.324** | **0.99** | **0.041** |
| Left putamen | 29 | -26 | -4 | -6 |  |  |  |
| Left pallidum | 12 | -24 | -6 | -4 |  |  |  |

Note: The interaction effects were determined using a 2 (Group: Parkinson’s Disease vs. Healthy Control) × 2 (Seed: Inter-effector vs. Effector) mixed ANCOVA.
